# Genome-wide association study for deoxynivalenol production and aggressiveness in wheat and rye head blight by resequencing 92 isolates of *Fusarium culmorum*

**DOI:** 10.1186/s12864-021-07931-5

**Published:** 2021-08-30

**Authors:** Thomas Miedaner, Andrea Vasquez, Valheria Castiblanco, Hilda Elena Castillo, Nora Foroud, Tobias Würschum, Willmar Leiser

**Affiliations:** 1 University of Hohenheim, State Plant Breeding Institute, Fruwirthstr. 21, 70599 Stuttgart, Germany; 2 Present Address: Department of Plant Cell Biology, Schwann-Schleiden Centre, Georg-August-University Goettingen, Julia-Lermontowa-Weg 3, 37077 Goettingen, Germany; 3 Present Address: International Center for Tropical Agriculture, Cali, Colombia; 4 Present Address: Hilda Elena Castillo, Instituto de Investigación Agropecuaria de Panamá, Panamá City, Panamá; 5 Agriculture and Agrifood Canada, Lethbridge Research and Development Center, P.O. Box 3000, Lethbridge, Alberta T1J 4B1 Canada; 6 Institute of Plant Breeding, Seed Science and Population Genetics, University of Hohenheim, 70599 Stuttgart, Germany

**Keywords:** *Fusarium culmorum*, Fusarium head blight, GWAS, Mycotoxins, Trichothecenes, *Tri* genes, *T. aestivum*, *S. cereale*, Whole-genome sequencing

## Abstract

**Background:**

*Fusarium culmorum* is an important pathogen causing head blight of cereals in Europe. This disease is of worldwide importance leading to reduced yield, grain quality, and contamination by mycotoxins. These mycotoxins are harmful for livestock and humans; therefore, many countries have strict regulatory limits for raw materials and processed food. Extensive genetic diversity is described among field populations of *F. culmorum* isolates for aggressiveness and production of the trichothecene mycotoxin deoxynivalenol (DON). However, the causes for this quantitative variation are not clear, yet. We analyzed 92 isolates sampled from different field populations in Germany, Russia, and Syria together with an international collection for aggressiveness and DON production in replicated field experiments at two locations in two years with two hosts, wheat and rye. The 30x coverage whole-genome resequencing of all isolates resulted in the identification of 130,389 high quality single nucleotide polymorphisms (SNPs) that were used for the first genome-wide association study in this phytopathogenic fungus.

**Results:**

In wheat, 20 and 27 SNPs were detected for aggressiveness and DON content, respectively, of which 10 overlapped. Additionally, two different SNPs were significantly associated with aggressiveness in rye that were among those SNPs being associated with DON production in wheat. Most of the SNPs explained only a small proportion of genotypic variance (*p*_*G*_), however, four SNPs were associated with major quantitative trait loci (QTLs) with *p*_*G*_ ranging from 12 to 48%. The QTL with the highest *p*_*G*_ was involved in DON production and associated with a SNP most probably located within the *Tri4* gene.

**Conclusions:**

The diversity of 92 isolates of *F. culmorum* were captured using a heuristic approach. Key phenotypic traits, SNPs, and candidate genes underlying aggressiveness and DON production were identified. Clearly, many QTLs are responsible for aggressiveness and DON content in wheat, both traits following a quantitative inheritance. Several SNPs involved in DON metabolism, among them the *Tri4* gene of the trichothecene pathway, were inferred as important source of variation in fungal aggressiveness. Using this information underlying the phenotypic variation will be of paramount importance in evaluating strategies for successful resistance breeding.

**Supplementary Information:**

The online version contains supplementary material available at 10.1186/s12864-021-07931-5.

## Background

*Fusarium* belongs to one of the most diverse and widely distributed fungal genera producing a broad variety of secondary metabolites [1]. It causes many economically important diseases in crops, the most devastating is Fusarium head blight (FHB). In Europe, FHB is caused by about 15 *Fusarium* species [2], where *F. graminearum* and *F. culmorum* are frequently isolated members [3–5]. They both cause stalk and ear rot in maize [6, 7] and seedling blight, foot rot, and head blight in small-grain cereals including wheat, barley, rye and triticale [8]. A common symptom of FHB is the pre-mature bleaching of infected spikelets. Kernels can be aborted completely when the ears are colonized during flowering. Even with low levels of infection, FHB diminishes grain quality and can reduce yield by nearly 50% [9]. In addition to yield loss, *F. culmorum* infection can be even more problematic since the fungus produces an array of mycotoxins, including type B trichothecenes, which are inhibitors of protein synthesis in plants and animals. The main trichothecenes reported in *F. culmorum-*infected cereals include deoxynivalenol (DON), nivalenol (NIV), and their acetylated derivatives [10], but in addition to these, each isolate can produce numerous other mycotoxins. For example, a single *F. culmorum* isolate was described that produced nine additional mycotoxins on wheat, one of which was zearalenone [11]. Crop mycotoxin contamination by *Fusarium* spp. is a worldwide problem and therefore strict regulatory limits are established in many countries regarding the amount of mycotoxins legally permitted in grains for food/feed usage and in processed food in the EU, USA, and Canada [10]. Consequently, *Fusarium* infection of cereals results in decreased market value or even the rejection of the grain.

Pathogenicity is the ability of the pathogen to infect a susceptible host [12]. If infection is caused by a gene-for-gene relationship, then the observed pathogenicity is a qualitative trait called ‘virulence’, determined by the presence or absence of resistance genes that give rise to plant immunity. Conversely, if the phenotype of pathogenicity represents a continuous distribution from low to high infection rate, it is a quantitative trait denoted as ‘aggressiveness’ [13]. In order to assess quantitative pathogenicity, symptom development and toxin production are often used [14, 15]. As a quantitative trait, aggressiveness is highly influenced by the environment [15], controlled by multiple genes and expected to be slowly evolving [14, 16]. Variation in aggressiveness within pathogen populations could lead to a gradual adaptation of the pathogen to resistant hosts in the long term [16, 17]. Since *F. culmorum* has a wide host range, it offers an ideal opportunity to unravel the hidden genetic basis of quantitative pathogenicity. Previous studies consistently reported a large variation in aggressiveness of *F. culmorum* [18–21]. Older studies reported several genes to be associated with aggressiveness on the basis of replacement mutants, among them are a secreted lipase *FgFGL1* [22]*,* other hydrolyzing enzymes [23] and ABC transporters in *F. culmorum* and *F. graminearum* [24, 25]. However, due to the large progress in transcriptomics and genomics in *F. graminearum*, many other potential genes and genomic regions have been identified [23, 26–29]. About 70 h after infection, mycotoxins trigger plant tissue necrosis [30]. Accordingly, the trichothecene pathway is a major factor that has been definitively and repeatedly attributed with *F. graminearum* aggressiveness, in particular the *Tri5* gene encoding the first pathway enzyme was reported in multiple studies to be linked with aggressiveness [31–34]. DON-nonproducing mutants of *F. culmorum* and *F. graminearum* cause reduced symptoms compared with wild-type isolates [31, 33, 35]. In either species, while they are still able to infect and cause symptoms on the inoculated spikelets, they do not spread within the head. Therefore, DON is a factor of aggressiveness rather than a pathogenicity factor. More recently, the trichothecene toxin gene cluster was found among 14 regions with distinct signatures of selection in North American *F. graminearum* populations [29] providing another evidence for the importance of the trichothecenes.

Twelve of the trichothecene genes in *F. graminearum* are clustered within a 25-kb region on chromosome II that is inherited as one core TRI cluster [36, 37]. Two other loci are involved: the single gene *Tri101* and the two-gene *Tri1-Tri16* locus, both located in unlinked chromosomal regions [10]. The presence of functional *Tri7* and *Tri13* genes is required for NIV-producing chemotypes in both *F. graminearum* [38] and *F. culmorum* [39]. The trichothecene cluster of the *F. culmorum* genome is structured similarly to that of *F. graminearum,* as shown by Schmidt et al. [1] in their analysis of a DON-producing *F. culmorum* isolate. In the meantime, however, NIV-producing isolates are increasing in frequency in several geographic regions, as has been reported for different *Fusarium* species [7, 40, 41]. For example, in the UK they are already dominating the *F. culmorum* population with a frequency of 75.1% compared to 15.0% in *F. graminearum* at least in maize [7]. Although DON seems to play an important role in the development of disease and aggressiveness, there appears to be a multitude of genes responsible for the broad genetic variation in aggressiveness of *Fusarium* spp. Indeed, several other genes have been described as aggressiveness factors in candidate-gene association studies in *F. culmorum* [21, 42] and in a whole-genome based association mapping study of *F. graminearum* [43].

The understanding of the physiological function of genes, chromosomal organization and related gene products or metabolites has been boosted by the arrival of next generation sequencing technologies, assisting to elucidate the genetic and molecular features that facilitate success of some fungal species as plant pathogens [29, 44, 45]. *F. graminearum* was the first member of cereal-infecting Fusaria whose complete genome assembly was provided [23], which has since been refined several times [46–48]. For *F. culmorum,* there are two genome sequences available, one from the Australian strain CS7071 [(PRJEB1738) [49], GenBank accession CBMH010000000], whose genome sequence is a fragmented assembly that belongs to the “Wheat Pathogen Genomes” project of the Bioplatforms Australia (https://downloads.bioplatforms.com/wheat_pathogens/) and the other from a United Kingdom field strain, UK99 [50]. The latter is a genome sequence of nearly 42 Mb, after removal of unknown bases just over 39 Mb, which is close to the genome size of *F. graminearum* strain PH-1 with 36.6 Mb [47]. The draft genome assembly of *F. culmorum* revealed four chromosomes using alignments to *F. graminearum* PH-1, but also yielded two small pseudomolecules concatenated by unplaced contigs > 2 kb (“chromosome 5”) and < 2 kb (“chromosome 6”) [50].

Although the full genome sequence of *F. culmorum* is available, to date only two genes have been associated with pathogenicity or aggressiveness according to the Pathogen-Host-Interaction database (http://www.phi-base.org/searchFacet.htm?queryTerm=Fusarium+culmorum): an ABC transporter and a ROGDI like leucine zipper domain regulating Trichothecene type B biosynthesis [24, 51]. Different methods are now available to further analyze the underlying mechanisms of the large quantitative variation in aggressiveness among strains. Comparative genomics enables the detection of genes responsible for basic pathogenicity across and within *Fusarium* species [29, 44, 45, 52]. Wang et al. [52] postulated a conserved two-speed genome from the resequencing of three Chinese strains of *F. graminearum* with the fast sub genome relating to host-pathogen interaction. High-throughput gene deletion studies have also proven useful in identifying genes essential for pathogenicity, e.g., [53, 54]. Neither of these approaches, however, are designed to search for genes specifically responsible for quantitative, within-species differences in aggressiveness or mycotoxin production. Therefore, we applied an association mapping that is widely used for studies of quantitative inheritance of key traits in crops [55]. Candidate-gene based association mapping revealed a few genes that could be associated with differences of aggressiveness in *F. culmorum* [21, 42], but only a small number of genes with annotated functions and identified by gene-deletion studies in the greenhouse are accessible.

To obtain a broader picture of the genetics of aggressiveness and DON production of this important fungal pathogen in its natural environment, we re-sequenced the genome of 92 isolates of *F. culmorum* and used 134,789 high quality single-nucleotide polymorphisms (SNPs) to conduct a genome-wide association study (GWAS). We thereby took advantage of the natural diversity produced by historical mutation events among field populations of *F. culmorum* sampled from different countries and phenotyped them in multi-environment trials. This study is unique since, until now, no resequencing study, e.g., [1, 29, 45] comprised phenotypic data on aggressiveness and DON content from field environments.

## Results

### Whole-genome data analysis identified 134,789 SNPs

After mapping the sequences from the 92 isolates against the reference genome, we identified 134,789 SNPs. The density of SNPs on *F. culmorum* chromosomes was 3.66 per kb on average (S2 Table) and varied from 2.96 to 5.19 SNPs per kb. SNP density for each chromosome is shown in S1 Fig. From these, 103,167 SNPs are transitions and 31,622 are transversions, representing a Ts/Tv ratio of 3.26 (S2 Fig). Based on predictions by SnpEff, most of the SNPs were found in upstream or downstream gene regions (38.7 and 38.5%, respectively), while 12.3% are located in transcripts, 4.8% in exons, 4.2% in intergenic regions and the remaining 1.4% in other regions such as splice sites and UTR regions. Taking into account the annotation of the genes, it was possible to predict that 56.8% of the identified SNPs in coding regions are silent, 42.5% of them are missense and 0.7% are nonsense changes. After removing SNPs with missing values > 5% and a minor allele frequency < 5%, 131,605 SNPs were used for further analyses.

### Phenotypic data analysis revealed large genotypic variance in aggressiveness

For DON content in wheat (DON-WH), the lowest concentration detected was 0.44 mg kg^− 1^ for FC50, which is in fact a NIV chemotype and the DON value might be a contamination, and the highest value of 20.7 mg kg^− 1^ was detected for isolate 9D22 (Table 1, S3 Table). The mean value for DON content was 12.3 mg kg^− 1^. Regarding aggressiveness on wheat (AGG-WH), the mean value was 15.5% with a minimum of 6.9% and a maximum of 19.5% for isolates S129 and S109, respectively. Values of aggressiveness in rye (AGG-RYE) ranged from 3.4% for isolate S129 to 27.9% for isolate FC95 with a mean of 15.7%. The entry-mean heritabilities were high for all traits, ranging from 0.85 to 0.89, thus providing a good starting point for association analysis.

**Table 1:** Phenotypic data and variance components for genotype (${\sigma}_G^2$), genotype-by-environment interaction (${\sigma}_{G\times E}^2$), error (${\sigma}_e^2$), and entry-mean heritabilities for aggressiveness in wheat (AGG-WH) and rye (AGG-RYE) and deoxynivalenol content in wheat (DON-WH). Estimates were calculated with arcsine square root transformed data

Parameter	DON-WH	AGG-WH	AGG-RYE
	mg kg^− 1^	(%)	(%)
Phenotypic data:^a^			
Minimum	0.40	6.90	3.40
Mean	12.3	15.5	15.7
Maximum	20.7	19.5	27.9
LSD _5%_	2.58	0.04	0.06
Variance components (× 10^− 3^):			
${\sigma}_G^2$	4.66^b^	1.16^b^	2.85^b^
${\sigma}_{G\times E}^2$	1.44^b^	0.75^b^	2.80^b^
${\sigma}_e^2$	9.01	1.37	0.86
Heritability	0.89	0.87	0.85

^a^ Backtransformed data

^b^ Significantly different from zero at the 0.001 level of probability

**Fig. 1:**
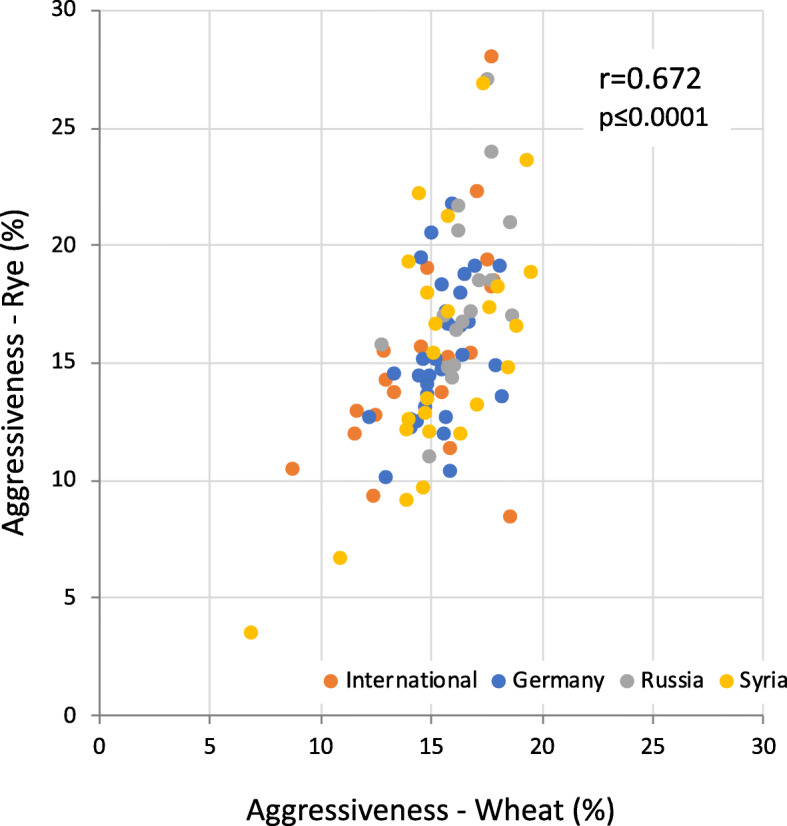
Relationship between wheat and rye aggressiveness calculated as BLUES with mean Fusarium head blight (FHB) rating of 92 *Fusarium culmorum* isolates across four environments; r = coefficient of correlation, p = probability of error

When color-coding the subpopulations in a scatterplot for aggressiveness on wheat and rye (Fig. 1), no clustering based on origin occurs. For example, the aggressiveness of the Syrian isolates spans the entire range of aggressiveness for both cereal species. In addition, the correlation of aggressiveness between rye and wheat is significant, but not complete across four environments.

The genotypic variance (${\sigma}_G^2\Big)$ as well as the genotype-by-environment interaction variance (${\sigma}_{G\times E}^2\Big)$ were significantly (*P* < 0.001) different from zero for all traits. The correlation between aggressiveness in wheat and DON production was significant (*r* = 0.77, *p* < 0.001) (S3 Fig). Furthermore, the correlation between aggressiveness in rye and aggressiveness or DON production in wheat were moderate and significant (*r*^*2*^ = 0.62 and *r*^*2*^ = 0.54, respectively, *p* < 0.001).

### Detection of population structure

Classical multidimensional scaling (MDS) of the distance matrix was performed and the resulting principal components revealed some degree of population structure (S4 Fig). Resulting clusters represent the geographical origin of the populations and therefore the population membership of the isolates. Particularly the Syrian population is grouped apart from the German and the Russian clusters. The first principal component explained 25.93% of the total genetic variance, the second, third and fourth an additional 8.67, 5.72 and 4.65%, respectively. Consequently, principal components were added to the mixed linear model in order to correct for population structure (S5 Fig).

### Identification of SNPs associated with DON production and aggressiveness

The association analysis resulted in the identification of 27 SNPs that were associated with DON production (Table 2).

**Table 2:** Detected QTL for deoxynivalenol production in wheat (DON-WH), aggressiveness in wheat (AGG-WH) and rye (AGG-RYE)

Trait	Marker	Chr.^**a**^	Position	p value	***p***_***G***_^***a***^		α-Effect^a^
			(bp)		Combined	Single	
DON-	FC1_1332589	I	1,332,589	6.75E-05	1.39	11.50	− 2.26
WH	FC1_7462132	I	7,462,132	6.64E-06	3.44	27.02	−3.02
	FC1_7624829	I	7,624,829	4.57E-05	0.57	25.50	−3.19
	FC1_7634754	I	7,634,754	5.78E-05	0.72	21.13	−3.29
	FC1_8372372	I	8,372,372	3.73E-05	0.22	27.96	−3.23
	FC1_11609824	I	11,609,824	1.76E-05	0.59	35.77	−4.00
	FC2_497121	II	497,121	6.52E-05	4.60	18.36	−2.13
	FC2_4071753	II	4,071,753	2.76E-05	3.57	40.39	−4.01
	FC2_4592052	II	4,592,052	2.65E-06	4.24	29.98	−2.65
	FC2_5570470	II	5,570,470	5.94E-07	0.46	28.64	−2.71
	FC2_5574549	II	5,574,549	2.51E-05	0.50	33.11	−4.02
	FC2_5575822	II	5,575,822	8.24E-05	0.47	20.05	−2.74
	FC2_5593616	II	5,593,616	7.90E-09	48.35	48.35	−4.39
	FC2_5761776	II	5,761,776	2.99E-05	2.15	21.33	−3.01
	FC2_6904196	II	6,904,196	5.18E-07	1.03	23.92	−3.14
	FC2_7111625	II	7,111,625	6.23E-05	0.33	21.17	−2.52
	FC2_8319916	II	8,319,916	2.03E-05	0.01	23.67	−2.93
	FC2_8367818	II	8,367,818	1.63E-05	0.27	20	−2.47
	FC2_8496198	II	8,496,198	1.01E-05	1.43	26.4	−2.48
	FC3_605481	III	605,481	2.97E-05	0.71	33.3	−3.75
	FC3_1553327	III	1,553,327	1.83E-06	0.86	24.36	−2.75
	FC3_6685196	III	6,685,196	3.84E-05	1.54	25.26	−3.31
	FC3_6792804	III	6,792,804	4.48E-07	3.37	34.66	−4.15
	FC3_7355239	III	7,355,239	1.35E-05	0.46	31.03	−3.57
	FC3_7367207	III	7,367,207	2.20E-05	0.13	25.66	−2.44
	FC4_4553334	IV	4,553,334	4.09E-05	0.13	30.47	−2.64
	FC4_4911411	IV	4,911,411	3.38E-05	1.59	18.03	−2.31
	**Total**				70.37		
AGG-	FC1_1332589	I	1,332,589	2.14E-05	0.14	13.01	−1.47
WH	FC1_7462132	I	7,462,132	3.40E-05	1.99	23.62	−1.76
	FC1_7863082	I	7,863,082	5.56E-05	0.29	20.96	−1.87
	FC1_8372372	I	8,372,372	9.89E-05	0.39	23.17	−1.84
	FC2_5279785	II	5,279,785	8.92E-05	0.01	13.87	−1.58
	FC2_5570470	II	5,570,470	8.74E-05	0.64	16.81	−1.37
	FC2_5593616	II	5,593,616	9.50E-06	1.33	29.70	−2.27
	FC2_5720089	II	5,720,089	5.84E-05	0.11	18.94	−1.86
	FC2_5761776	II	5,761,776	2.41E-07	33.38	33.26	−2.12
	FC2_5762236	II	5,762,236	1.69E-05	0.01	26.90	−1.56
	FC2_6904196	II	6,904,196	2.84E-06	12.30	23.28	−2.00
	FC3_605481	III	605,481	2.76E-05	0.50	25.66	−2.11
	FC3_720389	III	720,389	7.37E-05	0.62	27.62	−1.92
	FC3_6685196	III	6,685,196	8.29E-05	0.01	15.15	−1.63
	FC3_7344552	III	7,344,552	7.77E-06	5.72	31.48	−2.29
	FC4_1464595	IV	1,464,595	7.26E-05	1.50	15.85	−1.56
	FC4_3857293	IV	3,857,293	3.30E-05	3.59	23.33	−2.03
	FC4_4553334	IV	4,553,334	5.12E-05	1.90	27.54	−1.58
	FC4_6994632	IV	6,994,632	3.93E-05	1.89	18.85	−1.41
	FC4_7436319	IV	7,436,319	6.80E-05	1.07	19.75	−1.87
	**Total**				**53.31**		
AGG-	FC1_7624829	I	7,624,829	4.05E-05	17.18	17.69	−2.98
RYE	FC3_6792804	III	6,792,804	5.52E-05	5.66	18.03	−3.26
	**Total**				**21.40**		

^a^Chr. *Chromosome,* p_G_ proportion of genotypic variance explained by the QTL in percentage in a combined and single analysis, α-Effect allele substitution effect

**Fig. 2:**
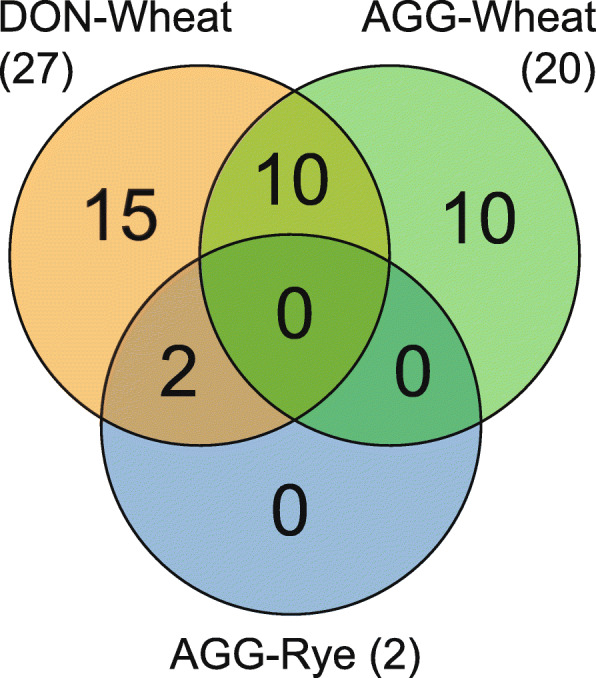
Venn diagram for the number of significantly associated SNPs for deoxynivalenol (DON) content in wheat and aggressiveness (AGG) in wheat and in rye

Two SNPs are significantly associated with the aggressiveness of *F. culmorum* in rye and 20 SNPs with aggressiveness in wheat. Interestingly, 10 of the SNPs that are significantly associated with DON-WH are also significantly associated with AGG-WH (Fig. 2) and the two SNPs that are associated with AGG-RYE are also associated with DON-WH. Surprisingly, there are no shared SNPs between aggressiveness in both hosts.

**Fig. 3:**
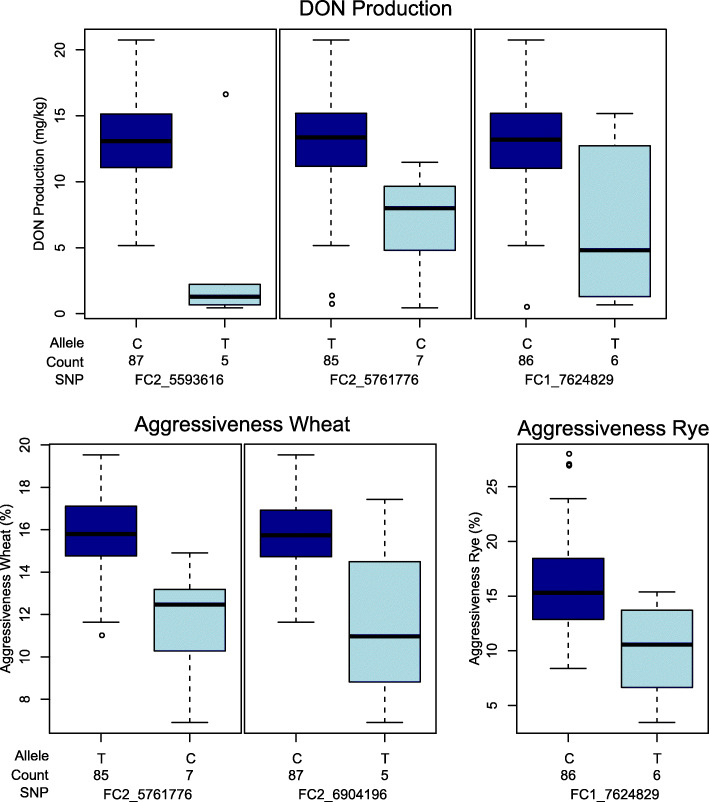
Boxplots of the SNPs explaining the largest proportion of genotypic variance for deoxynivalenol production in wheat and aggressiveness in wheat and rye with the two SNP alleles and the number of isolates bearing the respective allele (count); horizontal line within boxes = median, and ○ = outliers

The largest QTL identified for aggressiveness in rye, located on chromosome I, explains 17.2% of the genotypic variance. For aggressiveness in wheat, the largest QTL on chromosome II explains 33.4% of the genotypic variance and another, but linked QTL is the largest for DON concentration, explaining 48.4% of the genotypic variance (Table 2). When the allele frequency of the SNPs with the highest proportion of explained variance in the whole population was assessed, we found that most of the isolates carry the high aggressiveness allele (Fig. 3).

Two SNPs, FC2_6904196 and FC4_3857293, associated with aggressiveness in wheat are located within two genes, FCUL_06122 and FCUL_10689, and cause amino acid changes (Table 3). Furthermore, the SNP FC1_7462132 associated with aggressiveness and DON production in wheat is a splice region variant (intron variant). All mentioned protein sequences have an unknown function.

**Table 3:** SNPs that are significantly associated with aggressiveness (AGG-WH) and deoxynivalenol content (DON-WH) in wheat, explaining more than 2% of genotypic variance and located in gene sequences

Marker	Chr	Position	Trait	p_G_ (%)	Protein ID	Predicted function	Mutation type	Amino acid change
FC2_6904196	II	6,904,196	AGG-WH	12.30	FCUL_06122	Unknown	Missense_variant (Pro41Ser)	Pro41Ser
FC4_3857293	IV	3,857,293	AGG-WH	3.59	FCUL_10689	Unknown	Missense_variant (Val839Ala)	Val839Ala
FC1_7462132	I	7,462,132	DON-WH	3.44	FCUL_02320	Unknown	Splice region variant and intron variant	

**Fig. 4:**
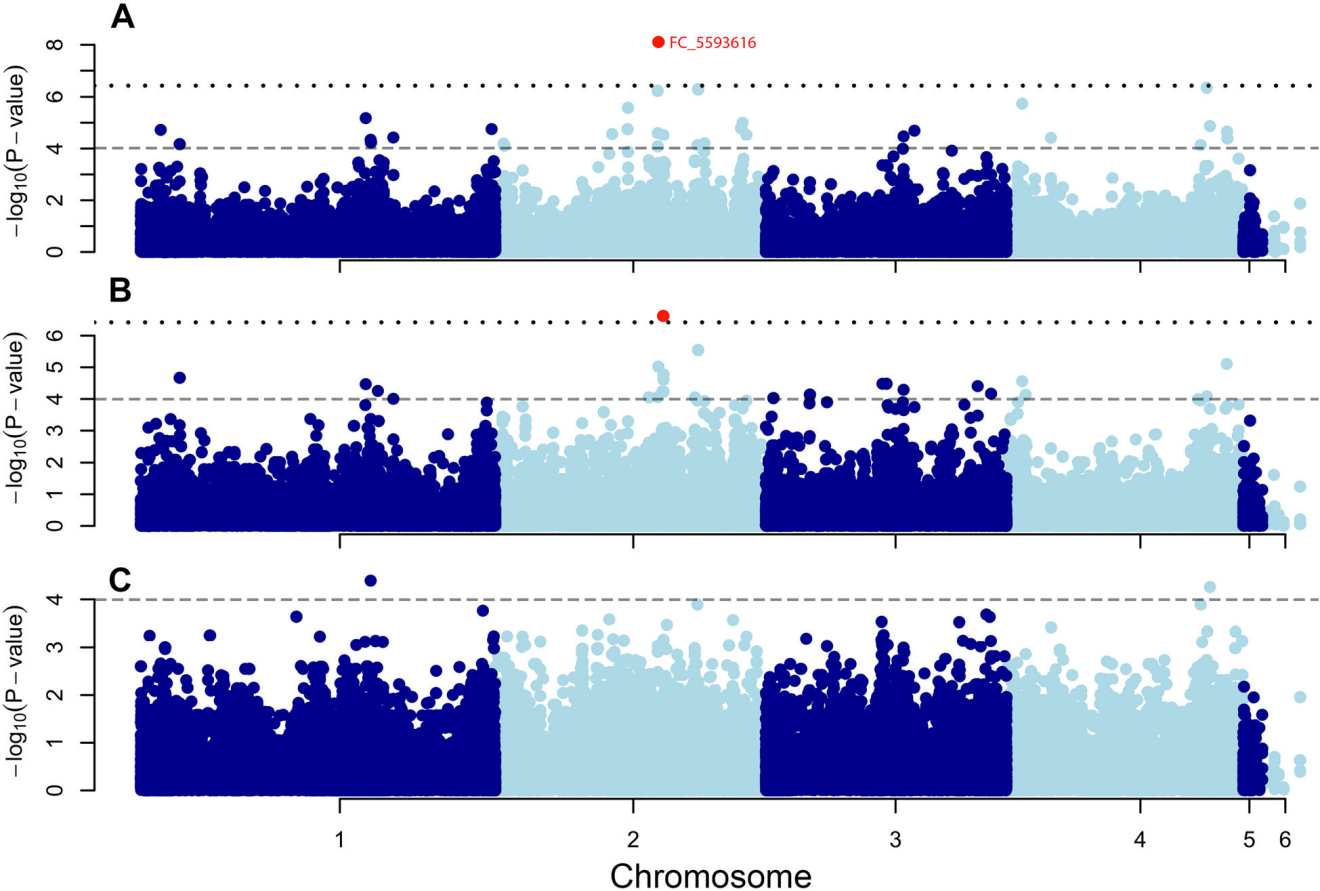
Manhattan plots of the genome-wide association study of (**A**) deoxynivalenol content in wheat, (**B**) aggressiveness in wheat, (**C**) aggressiveness in rye. The dotted line represents the Bonferroni significance threshold of *p* < 3,8 × 10^−7^ and the horizontal blue line the explorative significance threshold of *P*-value < 0.0001

**Fig. 5:**
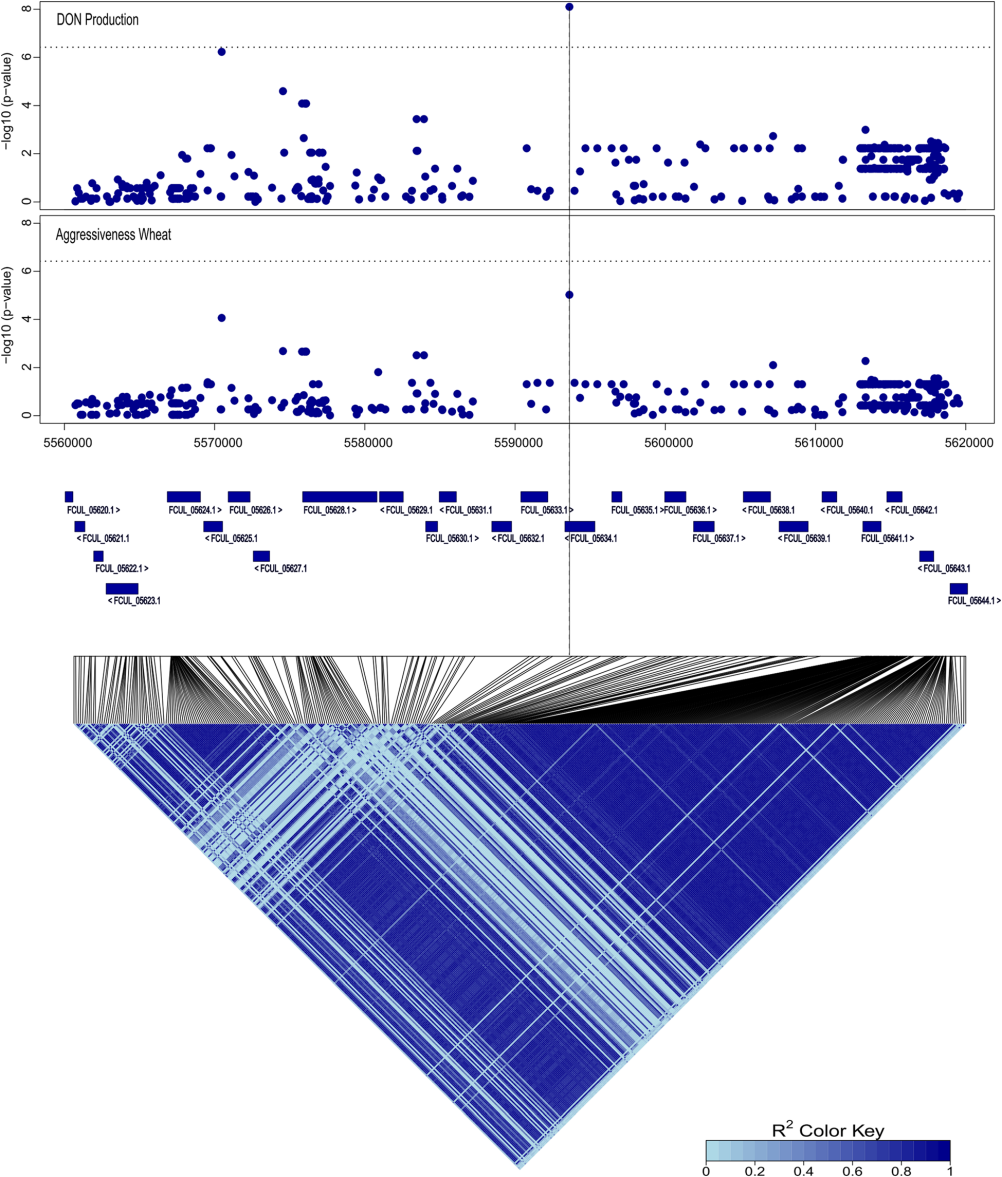
Manhattan plots for deoxynivalenol (DON) production and aggressiveness in wheat and of the genomic segment from 5.570.000 bp to 5.620.000 bp on chromosome 2 (49.145 bp, 467 SNPs) and results from the genome browser with the proposed genes in this region and LD block

We also identified a SNP (FC2_5593616) that is significantly associated with DON production and explains 48.4% of the genotypic variance. Although this SNP explains just 1.33% of the genotypic variance of aggressiveness in wheat, it is the only SNP with a significance value above the Bonferroni threshold (Fig. 4). This SNP is located in the gene FCUL_05634 that encodes for a Cytochrome P450 [56, 57] with a length of 520 amino acids. The translated sequence of this gene shares 95.7% of identity with the sequence of *Tri4* from *F. graminearum* and 96.9% with the respective protein from *F. pseudograminearum,* as confirmed by reciprocal blastp. Additionally, we identified the gene cluster for biosynthesis of trichothecenes in *F. culmorum* using the antiSMASH pipeline, and each of the core cluster candidate genes was confirmed by blastp with the *F. graminearum* sequences as query. The FCUL_05634 gene is the third gene of the core cluster and based on these data, we conclude that the gene FCUL_05634 is orthologous to the *Tri4* gene previously reported in other species [58]. Given that the gene cluster where the DON biosynthesis genes are located is in a linkage disequilibrium (LD) block (Fig. 5), the associated SNP could either represent an effect of the *Tri4* gene or be linked to any other gene within the cluster. It thus represents a QTL that contributes to aggressiveness and DON content of *F. culmorum*.

## Discussion

*Fusarium culmorum* is, besides *F. graminearum*, one of the most frequently occurring *Fusarium* species associated with Fusarium head blight disease in Europe [4, 5]. However, not much is known about the basis of its pathogenicity or aggressiveness. The availability of an annotated whole-genome assembly [50] opens new routes towards an understanding of aggressiveness. Association mapping is based on a diversity panel of individuals with complex relatedness that have captured historical recombination events during evolution [55]. To identify significant associations between genotypes and phenotypes, high-density genetic markers are necessary, because the method is based on linkage disequilibrium between markers and causative nucleotides. For the first time in *F. culmorum*, we have analyzed the whole-genome sequences of 92 isolates aiming for an association study with two important phenotypic features, aggressiveness and DON production.

### Large genetic variation of aggressiveness and DON production

The results of this study support the outcome of previous studies revealing large genetic variation of aggressiveness and DON production among *F. culmorum* isolates [18–20, 59]. The genetic variation was estimated statistically by partitioning the phenotypic variance measured in our field experiment across four location × year combinations (=environments) into the components of genotype, genotype-by-environment interaction, and error. The genetic component was the largest for all three traits (Table 1). The genotype-by-environment interaction, however, had a similar impact for aggressiveness in rye, but was smaller for the traits in wheat. The genetic causes for this large genetic variation in *F. culmorum* are not clear, because there is no sexual stage known in this fungal species. However, neutral molecular markers also revealed a large variation among 186 isolates [60]. This points to a recombining structure of *F. culmorum* [61], which may be explained by one or any of the following hypotheses: (1) cryptic variation by a sexual stage; (2) the teleomorph has become extinct; or (3) asexual variation, e.g. by heterokaryosis. The first two hypotheses are supported by the fact that *F. culmorum* has two transcribed mating type gene homologues [62, 63] occurring at similar frequencies in natural populations [61].

In our study, the aggressiveness in rye reached higher values than that in wheat. This does not reflect the reality when testing larger arrays of host genotypes [64]. From our previous knowledge that rye is generally less susceptible to FHB than wheat, we have chosen a highly susceptible rye genotype to get an optimal differentiation of the isolates.

### Several QTLs identified for aggressiveness and DON production

Because aggressiveness is inherited quantitatively, each gene contributing to this complex trait is expected to explain only a small proportion of the genotypic variance. This has been recently shown by candidate-gene based association mapping [21, 42] where the associated candidate genes had contributions to the genotypic variation varying from 6.05 to 16.05%. Moreover, Castiblanco et al. [42] attributed different candidate genes to aggressiveness of wheat and rye. Especially the gene cutinase (*CUT*) was significantly associated with aggressiveness towards rye but not towards wheat. This was attributed by the authors to the different composition of the cuticles of wheat and rye. Moreover, rye and wheat were overlapping only in 1 year (2015), in 2014 only wheat and in 2016 only rye was analyzed. This might have caused differences due to the highly significant genotype × environment interaction observed also in this study that is typical for the pathosystem. The complex genetic control underlying aggressiveness is corroborated by the finding of this study, that no QTL for aggressiveness were common between wheat and rye.

When the allele frequency of the SNPs was assessed in the whole population, we found that most of the isolates carry the high aggressiveness allele (Fig. 3). This might be related to the fact that the isolates used in this study were sampled from symptomatic heads of commercial wheat fields where low aggressiveness isolates are hardly found.

Two SNPs significantly associated with aggressiveness in wheat were found to be the proteins FCUL_06122 and FCUL_10689 with no predicted function in the *F. culmorum* genome. A blast of the genomic and the peptide sequences of both genes against the NCBI database and the UniProt database did not reveal for FCUL_06122 any more specific functions. The best hits, all on *Fusarium* species, also resulted in “uncharacterized protein”. For FCUL_10689 the best hits on UniProts were also unknown proteins, but in *Fusarium longipes, Fusarium sporotrichioides,* and *Fusarium vanettenii* (syn. *Fusarium solani* f. sp. *pisi*) peptide sequences were annotated as Transaldolase. Transaldolase (EC 2.2.1.2) was associated with the sugar and energy metabolism and one of the most abundant protein spots identified in *B. cinerea* mycelium [65]. Interestingly, a transaldolase-like protein (TAL 1) of *Cochliobolus heterostrophus* was identified as a *Hog1*-dependent gene playing an important role in protection against oxidative stress [66]. And *Hog-1* was significantly associated with aggressiveness and DON production in our candidate-gene association mapping of 100 *F. culmorum* isolates [21] of which 92 were included in this study.

### A large part of aggressiveness is explained by QTL responsible for DON production

The correlation between aggressiveness and DON production in wheat was significant, corroborating previous studies [21, 42, 67, 68]. Interestingly, all QTL associated with aggressiveness in rye and 10 out of 20 QTL associated with aggressiveness in wheat were also associated with DON content in wheat (Fig. 2). This points to a strong effect of DON on the infection process in the head. To date, however, cause and effect could not been separated. Gang et al. [18], for example, proposed that higher DON levels might just be the consequence of higher fungal biomass. When additionally estimating fungal biomass by measuring exoantigen content, the correlation between aggressiveness and DON content per unit fungal protein was non-significant [69]. On the other hand, strains with higher DON production were found to be more aggressive in some wheat genotypes [70, 71]. Whether this is related to the level of DON production or other genetic factors remains unknown. Importantly, while DON-nonproducing isolates are not impaired in very early infection events and form infection structures and induce the development of necrotic lesions [72], they are slower at developing the disease and are not able to grow outside the inoculation site [31, 68]. Thus, DON content might be important for host tissue proliferation [73, 74] until a threshold is reached in the plant tissue, where more DON does not necessarily lead to more symptoms.

Results of this association study strongly promote an important role of DON content for aggressiveness. The SNP FC2_5593616, explaining 48.4% of the total genotypic variation in DON content and as the only SNP exceeding the conservative Bonferroni threshold, is located in a gene with a very high identity (95.7%) with the published sequence of the *Tri4* gene of *F. graminearum*. This gene encodes a multifunctional cytochrome P450 monooxygenase for different oxygenation and hydroxylation steps in the trichothecene pathway leading to DON [58] and is, therefore, an indispensable component for trichothecene biosynthesis [75]. Alternatively, our most prominent SNP FC2_5593616 could also be in linkage disequilibrium with another gene from this cluster and not directly represent *Tri4*, but it is nonetheless an important factor in DON production having a high impact on aggressiveness. However, our study also shows that the large differences in aggressiveness among *F. culmorum* isolates most probably involve a multitude of quantitative loci of which *Tri4* is only one, although an important one. Accordingly, Talas et al. [43] found 26 and 17 genes significantly associated with aggressiveness and DON production, respectively, among 119 isolates of *F. graminearum*, each explaining 8 to 24% of the genotypic variance. As in our study, most genes detected did not have a known function.

## Conclusions

The high phenotypic variation of aggressiveness and DON production of *F. culmorum* is most likely caused by a multitude of genes of whom those of the DON pathway have an important function. Among them, we could show here that the *Tri4* gene or a gene in close linkage to *Tri4* explained nearly half of the total genotypic variation. More functional studies will be needed in future to further decipher the molecular mechanisms underlying their associations with fungal aggressiveness.

## Methods

### Fusarium culmorum isolates

A total of 92 isolates of *F. culmorum* collected from three different geographic origins, were used (S1 table). A group of 33 isolates were collected in three commercial fields in Germany, 18 isolates originated from a Russian field population and a transect population of 25 isolates was collected in Syria. Additionally, 19 isolates from the international collection of *F. culmorum* sampled from 12 different countries were included. These isolates were available in the rye group of the State Breeding Institute at the University of Hohenheim and came from single-spore isolation. Isolates were stored as colonized agar plugs in sterile water in 2 ml Eppendorf tubes at + 6 °C.

### Phenotypic data analyses of *F. culmorum* aggressiveness

Aggressiveness of *F. culmorum* in wheat and rye, and DON content in wheat were assessed. Raw phenotypic data from previous field studies were used as described in detail by Castiblanco et al. [21, 42]. Briefly, two host species with one cultivar each were used for inoculation: a winter wheat (*T. aestivum* L.) cultivar that is moderately susceptible (“Inspiration”, KWS LOCHOW SE, Bergen, Germany) and a highly susceptible, homogeneous rye single cross (*S. cereale* L., “L2177-P × L2184-N”, HYBRO Saatzucht GMBH & Co., KG, Schenkenberg, Germany). Inoculations and phenotyping were done at two locations: Hohenheim (HOH, 48° 42′ 54″ N, 9° 12′ 58″ E, 400 m) and Oberer Lindenhof (OLI, 48° 28′ 25″ N, 9° 18′ 12″E, altitude 700 m) in Germany in 2 years (2014 and 2015 for wheat as host and 2015 and 2016 for rye), which is equivalent to four environments (=location-year combinations).

Seeds were grown in two-row plots (rye) or three-row plots (wheat), respectively, with 1 m length and a row distance of 0.21 m. To decrease the drifting or secondary spore dispersal and avoid possible interference among plots, a chessboard-like design was used to arrange the entry plots that were bordered by long-strawed rye in the rye experiment and triticale (x*Triticosecale*) in the wheat experiment. Plots were sown with 220 kernels m^− 2^ resulting in a homogeneous dense plant stand. Experiments were designed following an incomplete block design (alpha design) with two replications per environment and an incomplete block size of 10.

Inoculum of all isolates was prepared in shaking liquid cultures according to the procedure of Reid et al. [76] and aliquots frozen at -80 °C. Before application, the samples were thawed in water at about 20 °C. Inoculation was performed by conidiospores in a dose of approximately 100 ml suspension per square meter and a concentration of 2 × 10^5^ conidiospores ml^− 1^. Inoculum for each isolate was sprayed onto the heads using a hand atomizer with constant air pressure of 3 bar produced by a tractor to ensure full coverage of all heads of the plot with the same dosage. All plots flowered simultaneously, because only one homogeneous rye or wheat genotype was used. This allowed inoculation and ratings for all plots at the same dates per location.

Aggressiveness values came from the arithmetic mean from at least three symptom ratings taken over time. The ratings started with the beginning of symptom development and were continued at 3- to 5-day intervals until the beginning of yellow ripening stages. Ratings represented the percentage of infected spikelets per ear (severity) and the percentage of infected ears per plot (incidence) in a single value from 0 to 100% [21, 42]. For determining DON content, wheat plots were harvested by hand at full ripening, carefully threshed in a single-head thresher (Walter-Wintersteiger, Austria) and cleaned with reduced wind speed. Cleaned wheat grain was ground in a commercial laboratory mill with a sieve size of 1 mm. The coarse meal was analyzed by a commercially available immunotest (Ridascreen FAST DON®, R-Biopharm AG, Darmstadt, Germany).

Statistical analyses of phenotypic data were done using the software R [77], outliers were detected in each environmental dataset with the BH-MADR method (Bonferroni–Holm with re-scaled MAD standardized residuals) suggested by Bernal-Vasquez et al. [78] and deleted accordingly. All data was arcsin transformed to meet the prerequisite of normality. Entry-mean heritabilities were computed as suggested by Piepho and Möhring [79]. The following mixed model was used to obtain the BLUEs (best linear unbiased estimators) for each isolate:

$$ \mathrm{G}+\mathrm{E}+\mathrm{G}\times \mathrm{E}+\mathrm{R}+\mathrm{B} $$

where G, E, R and B denote genotype, environment, replication and block, respectively, and G, E and R were modeled as fixed effects and G × E and B as random effects. R was modelled as nested within E, and B nested within R. Host (wheat or rye) was not considered in the model, given that pathogen fitness could depend on the host and since plant pathogens may use different strategies to infect different hosts [31]. Instead, we compared the outcome of our association mapping from the two hosts. The variance components were estimated using restricted maximum likelihood (REML) method and their significance was assessed by comparing the models using Likelihood Ratio test [80]. Pearson’s correlation (r^2^) between traits was calculated based on the BLUEs. The software package ASReml-R 3.0 [81] was used to fit the linear mixed models. All plots, except the local GWAS plot (see below), were created using basic R functionalities [77].

### DNA extraction, whole-genome resequencing, annotation and SNP calling

Plugs from each isolate were cultivated on PDA plates during 5 days at 20 °C. From each isolate, 200 mg of mycelia was scraped off from several plates with a sterile spatula and dried out with silica gel for 1 week. DNA extraction was performed following the cetyltrimethylammonium bromide (CTAB) method. DNA samples were run on a 1% agarose gel to check DNA stability and integrity with a O’GeneRuler 1 kb DNA ladder (Thermo Scientific) and the 23 kb DNA Molecular Weight Marker II (Sigma–Aldrich). DNA quality was measured with nanodrop, and quantity was measured with QuantiFluor® dsDNA System (Promega). Finally, the DNA was concentrated to 50 ng/μl. DNA samples were sent to Novogene Corporation (Beijing, China) where Illumina libraries were prepared for whole-genome sequencing using a Illumina HiSeq X with 150 bp paired-end sequencing. The resulting sequencing libraries had an average insert size of 300 bp.

Pair-end reads from all isolates were mapped to the reference genome with the Burrows-Wheeler Aligner software package (BWA, release 0.7.17) [82] using the MEM algorithm. As a reference genome, we used the draft assembly of *F. culmorum* strain UK99 that has almost 42 Mbp and consists of the 4 *F. culmorum* chromosomes plus two scaffolds of concatenated sequences that could not be assigned to the chromosomes [50]. The NGSEP software package [83] was used to identity SNPs, small indels, and structural variants in the assembled sequences, to make the functional annotation of each variant and to genotype each sample. Only bi-allelic SNPs with a quality call score larger than 40 (Phred scale), a minor allele frequency (MAF) of ≥0.05 and a minimum coverage of 95% of genotyped individuals were kept after filtering. Descriptive statistics such as SNPs density and position in *F. culmorum* chromosomes were calculated using the SnpEff tool [84] and vcftools [85] and mapping metrics were calculated with BBMap aligner [86]. Upstream and downstream gene region size was set to 5 kb. Around 9 millions of pair-end reads on average per each isolate were obtained after sequence quality check. From these reads, 91.6% were successfully aligned to the reference genome of *F. culmorum* strain UK99 covering an average of 87.7% of it, with a minimum of 81% and a maximum of 91.6%. The average sequencing depth was 33X and the mean insert length was 287.8 bp and ranged from 2 up to 636.3 kb. During the filtering process, SNPs that were mapped to repetitive sequences and copy number variations were excluded. Genotypic quality control of the final SNPs was performed using the R package GenABEL [87].

### Genome-wide association analysis

Given that isolates coming from different geographic regions were used for this study, stratification was assessed by principal component analysis with the R package GenABEL [87]. For this, a genomic relationship matrix between all pairs of individuals was computed and from this a distance matrix was constructed. Subsequently, a classical multidimensional scaling of the distance matrix was performed to obtain the principal components analysis of genetic variation.

Association analysis between SNP markers and traits was done with GenABEL [87], using a linear mixed model that included four principal coordinates for wheat and two for rye and a kinship matrix to adjust for possible population stratification. To control for multiple testing, Bonferroni-corrected significance threshold and, in addition, an explorative threshold of *p* < 0.0001 were used. All significantly associated markers of a trait were fitted into a linear model in the order of the strength of their association, i.e. with the most strongly associated marker fitted first [88]. The proportion of genotypic variance explained by all significantly associated SNPs was estimated as the ratio of the adjusted R^2^ of this linear model to the heritability of the trait ${p}_G=\frac{R_{adj}^2}{h^2}$ [89]. The proportion of genotypic variance explained by each single SNP was likewise derived from the sums of squares from this model (*p*_*G-combined*_). In addition, we also calculated the proportion of explained genotypic variance of each marker when fitted singly in a linear model (*p*_*G-single*_). The combination of the two values allows to draw conclusions whether significantly associated markers from one genomic region identify the same QTL, as the joint fit of all significantly associated markers corrects for collinearity among them. The additive effect (α-effect) of each individual significant SNP was derived from a linear model in which just the respective SNP was considered. The local GWAS plot was created using the R package LDheatmap [90].

To analyze the sequences that harbor the significantly associated SNPs, sequences of 100 bp around the SNPs were extracted with BEDTools [91] and genomic position and allele variation were confirmed in the *F. culmorum* genome. Translated sequences were compared with the protein sequences of closely related species. For the identification of secondary metabolite biosynthesis gene clusters in the whole genome of *F. culmorum* the web server antiSMASH 3.0 was used [92].

## Supplementary Information

**Additional file 1: Table S1.***Fusarium culmorum* isolates used in this study.

**Additional file 2: Table S2.** Number of SNPs identified and SNP density in each chromosome.

**Additional file 3: Table S3.** Best linear unbiased estimates (BLUES) for aggressiveness in wheat (AGG.WHEAT), aggressiveness in rye (AGG.RYE), and deoxynivalenol content in wheat (DON-WHEAT) for all tested isolates across locations and years.

**Additional file 4: Figure S1.** SNP density along the four chromosomes of *F. culmorum*. Histogram of variants per 100 Kb.

**Additional file 5: Figure S2.** SNP count across all the samples of *F. culmorum* classified by the mutation type.

**Additional file 6: Figure S3.** Frequency distritbution, phenotypic correlation coefficients and scatter plots for and between the evaluated traits for 92 isolates: Aggressiveness in wheat (AGG-WH) and in rye (AGG-RYE) and deoxynivalenol production in wheat (DON-WH). *** Significantly different from zero at 0.001 level of probability.

**Additional file 7: Figure S4.** Scatterplot of the first two principal components (PC) of 92 isolates of *F. culmorum* originating from Germany, Syria, Russia and isolates from the international collection. The proportion of explained variance is shown in brackets at the corresponding axes.

**Additional file 8: Figure S5.** Quantile-quantile plots (QQ-plots) for the aggressiveness in wheat (AGG-WH) and in rye (AGG-RYE) and deoxynivalenol production in wheat (DON-WH) showing the relation between expected and observed *P* values (−log_10_) for all SNPs after GWAS analysis adjusted for population stratification; Genomic inflation factor λ is given. The straight black diagonal lines represent the values under the null hypothesis of no association.

## Data Availability

All details on the analyzed isolates can be found in S1 Table, all phenotypic data are available as best linear unbiased estimates (BLUES) in the S3 Table (Supplementary Information). Sequencing data generated and analyzed during the current study are available in the NCBI (Bethesda, Maryland USA) database as BioProject ID PRJNA749912 [http://www.ncbi.nlm.nih.gov/bioproject/749912, release at Aug 08, 2021].

## Abbreviations

AGG: Aggressiveness
BLUE: Best linear unbiased estimator
DNA: Deoxyribonucleic acid
DON: Deoxynivalenol
FC: *Fusarium culmorum*
FHB: Fusarium head blight
GWAS: Genome-wide association study
NIV: Nivalenol
PDA: Potato dextrose agar
QTL: Quantitative trait locus
SNP: Single nucleotide polymorphisms
*Tri*: Trichothecene gene
WH: Wheat
