# Supplementary material for: Genome-wide association study for deoxynivalenol production and aggressiveness in wheat and rye head blight by resequencing 92 isolates of Fusarium culmorum
Source: BMC Genomics. 2021 Aug 30;22:630. doi: 10.1186/s12864-021-07931-5 (PMC8404269; doi:10.1186/s12864-021-07931-5)
Supplement: Supplementary file 2 — Additional file 2: Table S2. Number of SNPs identified and SNP density in each chromosome. [file 12864_2021_7931_MOESM2_ESM.pdf]

Table S2: Number of SNPs identified and SNP density in each chromosome.

Chromosome	Length	Variants	SNP/kb
1	11,803,652	34,981	2.96
2	9,183,391	47,636	5.19
3	7,941,633	27,915	3.52
4	7,957,372	23,696	2.98
(5)	3,208,310	485	0.15
(6)	1,834,517	76	0.04
**Total**	**41,928,875**	**134,789**	
