# Supplementary figures and images for: Genome-wide association study for deoxynivalenol production and aggressiveness in wheat and rye head blight by resequencing 92 isolates of Fusarium culmorum
Source: BMC Genomics. 2021 Aug 30;22:630. doi: 10.1186/s12864-021-07931-5 (PMC8404269; doi:10.1186/s12864-021-07931-5)

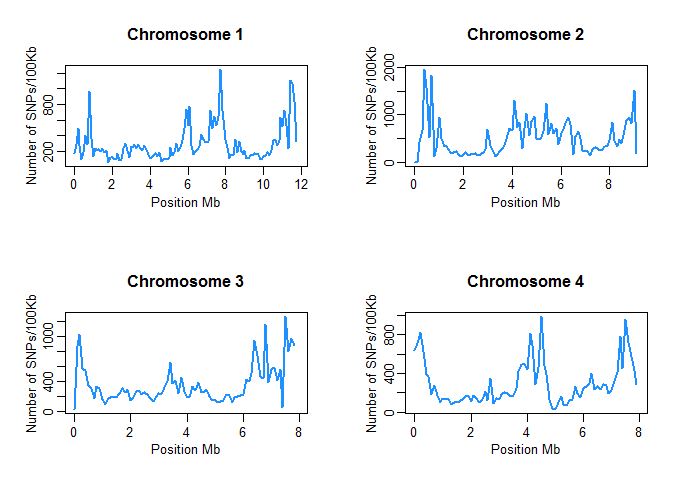

Supplement: Supplementary file 4 — Additional file 4: Figure S1. SNP density along the four chromosomes of F. culmorum. Histogram of variants per 100 Kb. [file 12864_2021_7931_MOESM4_ESM.png]

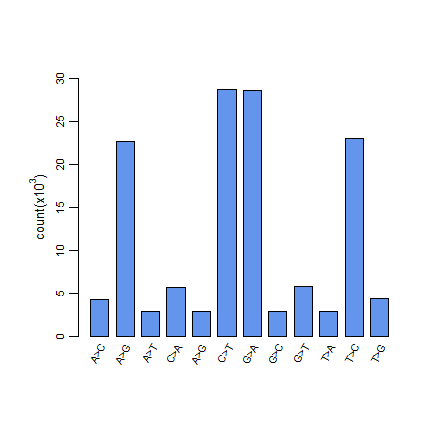

Supplement: Supplementary file 5 — Additional file 5: Figure S2. SNP count across all the samples of F. culmorum classified by the mutation type. [file 12864_2021_7931_MOESM5_ESM.png]

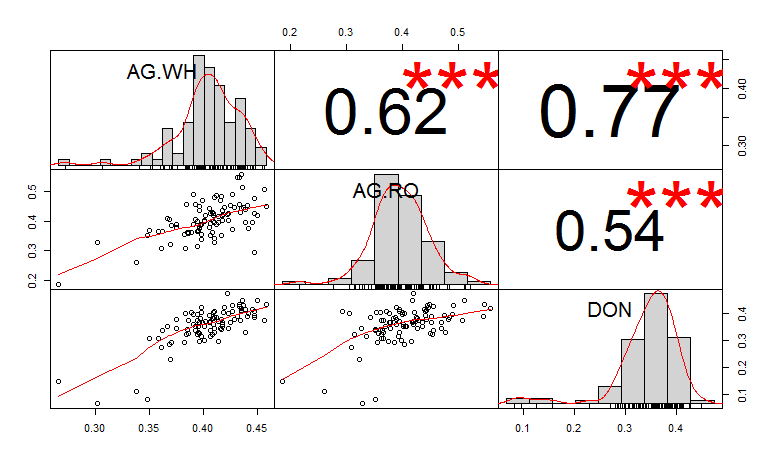

Supplement: Supplementary file 6 — Additional file 6: Figure S3. Frequency distritbution, phenotypic correlation coefficients and scatter plots for and between the evaluated traits for 92 isolates: Aggressiveness in wheat (AGG-WH) and in rye (AGG-RYE) and deoxynivalenol production in wheat (DON-WH). *** Significantly different from zero at 0.001 level of probability. [file 12864_2021_7931_MOESM6_ESM.png]

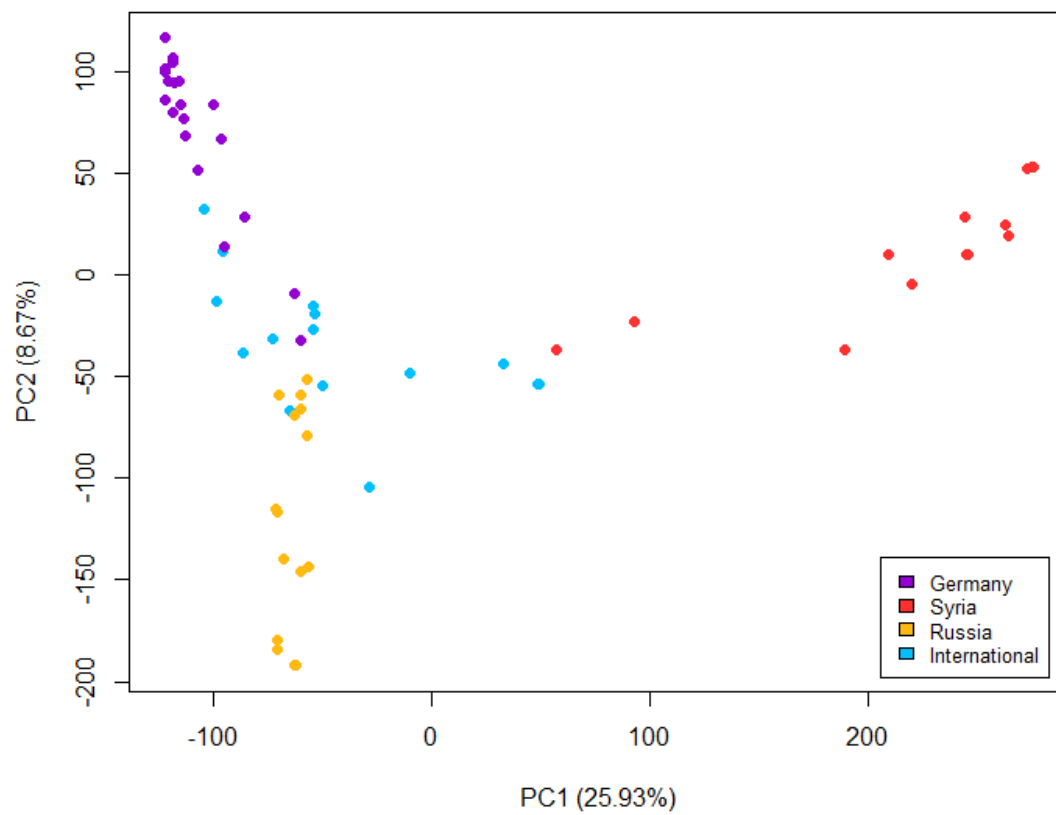

Supplement: Supplementary file 7 — Additional file 7: Figure S4. Scatterplot of the first two principal components (PC) of 92 isolates of F. culmorum originating from Germany, Syria, Russia and isolates from the international collection. The proportion of explained variance is shown in brackets at the corresponding axes. [file 12864_2021_7931_MOESM7_ESM.pdf]

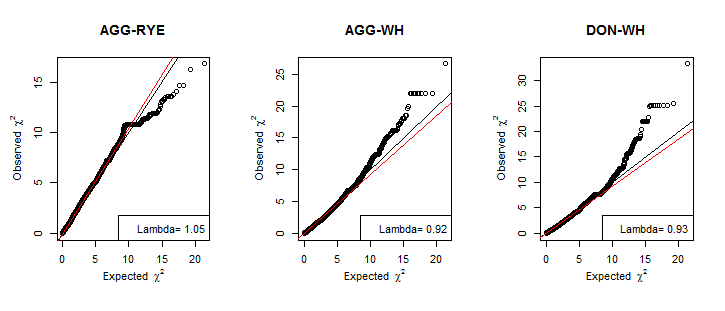

Supplement: Supplementary file 8 — Additional file 8: Figure S5. Quantile-quantile plots (QQ-plots) for the aggressiveness in wheat (AGG-WH) and in rye (AGG-RYE) and deoxynivalenol production in wheat (DON-WH) showing the relation between expected and observed P values (−log10) for all SNPs after GWAS analysis adjusted for population stratification; Genomic inflation factor λ is given. The straight black diagonal lines represent the values under the null hypothesis of no association. [file 12864_2021_7931_MOESM8_ESM.png]
